# Performance of large language models for extracting clinical data from breast cancer pathology reports: a systematic review

**DOI:** 10.1038/s41746-026-02789-x

**Published:** 2026-05-22

**Authors:** Ravi Shankar, Vahul Sundar, Ziyu Goh, Pei Yi Sin, Ern Yu Tan

**Affiliations:** 1 Clinical Research & Innovation Office, Tan Tock Seng Hospital, National Healthcare Group, Singapore, Singapore; 2 Yong Loo Lin School of Medicine, National University of Singapore, Singapore, Singapore; 3 Department of General Surgery, Tan Tock Seng Hospital, Singapore, Singapore; 4 Lee Kong Chian School of Medicine, Nanyang Technological University, Singapore, Singapore; 5 Institute of Molecular and Cell Biology, Agency for Science, Technology and Research, Singapore, Singapore

**Keywords:** Cancer, Computational biology and bioinformatics, Health care, Medical research, Oncology

## Abstract

Breast cancer pathology reports contain critical clinical information, yet manual extraction of structured data remains resource-intensive and error-prone. Large language models (LLMs) offer promising automated approaches, but no systematic synthesis examines their performance specifically for breast cancer pathology report processing. Following PRISMA guidelines, we searched seven databases from inception to December 2025, with two reviewers independently screening studies and extracting data. Methodological quality was assessed using PROBAST + AI and reporting completeness using TRIPOD + AI. Nine studies met inclusion criteria, evaluating over 30 distinct LLM architectures across datasets totaling approximately 14,161 reports. Best-performing models achieved study-specific accuracy ranging from 87.7% to 97.4%, though figures are not directly comparable across studies due to differences in task formulation, target data elements, and evaluation metrics. PROBAST + AI assessment found 55.6% of studies at low concern/risk across all domains, with the Outcome domain showing greatest variability. TRIPOD + AI revealed gaps in fairness reporting, open science practices, and patient/public involvement. LLMs demonstrate promising performance approaching human-level accuracy, but methodological quality varies, with key concerns regarding reference standard development, limited external validation, and inadequate fairness reporting.

## Introduction

Breast cancer pathology reports represent the definitive documentation of tumor characteristics essential for clinical decision-making, staging, treatment planning, and prognostication. These reports contain detailed information about tumor size, grade, histologic type, margin status, lymph node involvement, biomarker expression including estrogen receptor, progesterone receptor, and HER2 status, and numerous other features that collectively determine therapeutic approaches and patient outcomes^1^. The complexity and clinical importance of pathology reports have increased substantially with advances in molecular characterization, immunohistochemistry panels, and precision medicine approaches requiring granular documentation of tumor biology^2^. However, this wealth of information typically exists in unstructured narrative format, creating significant barriers for systematic data utilization in clinical care, quality monitoring, and research applications^3^.

The challenge of extracting structured data from pathology reports has profound implications for cancer care delivery and research. Manual abstraction remains the predominant approach in many settings, requiring trained personnel to read reports and enter data into structured databases for cancer registries, clinical trials, quality metrics, and research studies^4^. This process is time-consuming, expensive, and prone to inter-abstractor variability, with studies documenting error rates ranging from five to twenty percent even among experienced abstractors^5,6^. The volume of pathology reports generated annually compounds these challenges, with millions of breast cancer pathology reports produced globally requiring processing for various clinical and research purposes.

Natural language processing (NLP) approaches to pathology report extraction have evolved significantly over the past decade, progressing from rule-based systems through traditional machine learning to deep learning methods^7^. Early rule-based systems relied on pattern matching and regular expressions to identify specific data elements, achieving reasonable performance for well-defined fields but struggling with narrative complexity and institutional variations in reporting styles^8^. Traditional machine learning approaches including support vector machines and conditional random fields improved flexibility but required extensive feature engineering and domain expertise. The emergence of deep learning, particularly recurrent neural networks and early transformer models, demonstrated improved performance but still required substantial task-specific training data and customization for each extraction target^9^.

Several parallel research traditions in clinical information extraction also warrant consideration. First, structured extraction from pathology reports has been extensively studied through named entity recognition combined with relation extraction to produce subject–predicate–object triples (e.g., ‘tumor–hasGrade–G3’), a paradigm conceptually distinct from document-level classification and employing architectures such as BiLSTM-CRF pipelines and early BERT-based joint extraction models^10–14^. Second, ontology-guided extraction grounded in established vocabularies including SNOMED CT, ICD-O-3, and the College of American Pathologists synoptic reporting protocols has demonstrated improved consistency and reduced hallucination in structured output generation^15^. Third, consistency checking and post-hoc verification of extracted outputs against medical knowledge bases represents an active area of quality assurance research in clinical NLP pipelines^16^. These complementary approaches provide important context for evaluating the incremental contribution of LLM-based methods and highlight challenges, particularly around output consistency verification, that remain relevant regardless of model architecture.

Large language models (LLMs) represent a paradigm shift in clinical NLP through their ability to leverage massive-scale pretraining on diverse text corpora followed by task-specific fine-tuning or even zero-shot application^17^. Models such as BERT and its variants, GPT series, and specialized biomedical models like BioBERT and ClinicalBERT have demonstrated remarkable capabilities across various clinical NLP tasks^18^. For pathology report processing, LLMs offer several potential advantages including ability to handle varied report formats and terminology without extensive rule development, capacity to extract multiple data elements simultaneously, understanding of clinical context and implicit relationships, and potential for few-shot learning reducing annotation requirements^19^. These capabilities suggest transformative potential for automating pathology report processing at scale.

Despite enthusiastic adoption of LLMs for clinical NLP tasks, systematic evidence regarding their performance specifically for breast cancer pathology reports remains fragmented. Individual studies report varying performance metrics using different datasets, evaluation methods, and extraction targets, limiting ability to draw generalizable conclusions^9^. Questions persist regarding optimal model architectures for pathology report processing, training data requirements, performance variation across different data elements, generalization across institutions and reporting styles, and implementation considerations for clinical deployment. The rapid evolution of LLM architectures and training approaches further complicates the landscape, with new models and methods emerging faster than comprehensive evaluation can occur. This systematic review addresses these knowledge gaps by synthesizing available evidence on LLM performance for breast cancer pathology report extraction, providing critical guidance for clinical implementation and research priorities.

## Results

### Study selection

**Fig. 1:**
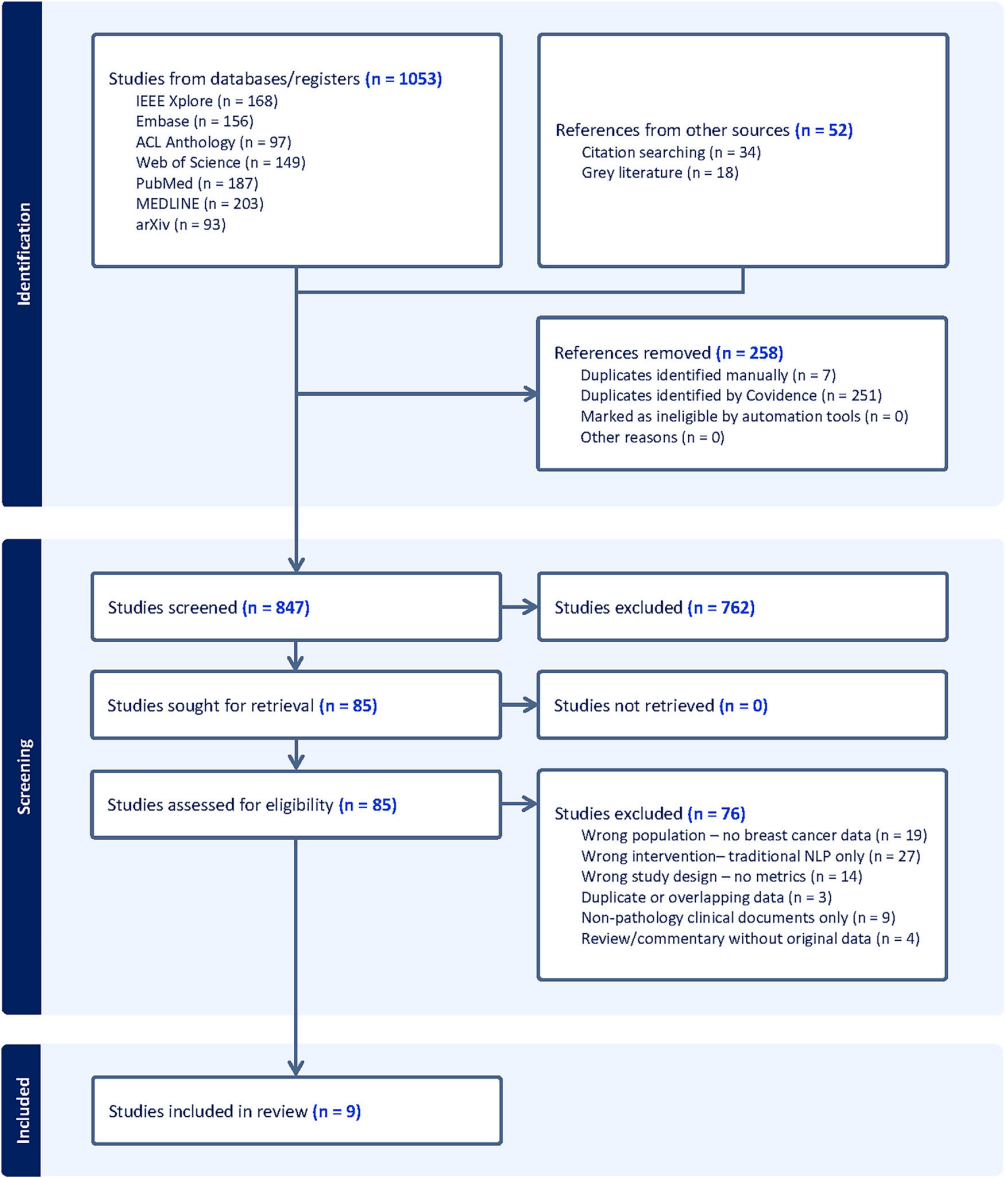
PRISMA flow diagram illustrating the study identification, screening, and selection process. Database searches identified 1053 records from seven sources including IEEE Xplore, Embase, ACL Anthology, Web of Science, PubMed, MEDLINE, and arXiv, with 52 additional references from citation searching and gray literature. After removal of 258 duplicates, 847 records were screened, 85 full-text articles were assessed for eligibility, and nine studies met all inclusion criteria. Common reasons for full-text exclusion are detailed in the figure. Created using the PRISMA 2020 flow diagram template (Page et al.^16^).

The database search identified 847 records after duplicate removal. Title and abstract screening excluded 762 records, with 85 proceeding to full-text review. Following full-text assessment, nine studies met all inclusion criteria and were included in the final synthesis^19–27^. Common reasons for exclusion included absence of breast cancer-specific data, use of traditional NLP methods without LLM components, lack of quantitative performance metrics, and processing of non-pathology clinical documents. The complete screening and study selection process is shown in the PRISMA flow diagram in Fig. 1. No amendments were made to the registered protocol (PROSPERO CRD420251012298) during the conduct of this review.

### Study characteristics

The nine included studies were published between 2023 and 2025, reflecting the recent emergence of LLM applications in clinical NLP. Geographic distribution included five studies from the United States^20,22,23,25,27^, two from Canada^19,24^, one from South Korea^21^, and one from Germany^26^. The German study (Bartels et al.^26^) examined a mixed oncological cohort of 522 reports, of which ~7% (*n* ≈ 37) were breast cancer cases. This study met eligibility criteria because performance metrics for individual cancer types, including breast cancer, were extractable from the reported results. However, we note that breast-cancer-specific performance could not be disaggregated from overall cohort performance for all metrics, and the small breast cancer subsample limits the precision of estimates attributable to this cancer type. While this study provided valuable evidence on RAG-enhanced extraction in a non-English setting, its breast-cancer-specific performance estimates should be interpreted with caution given the small subsample size. Study settings ranged from single academic medical centers to multi-institutional collaborations and population-based registries. Sample sizes varied considerably, from 53 reports in external validation subsets^27^ to 2931 patients in the largest cohort^21^. Report types included surgical pathology, core biopsy, synoptic reports, and scanned historical documents. Table 1 presents detailed study and dataset characteristics.

**Table 1:** Study and dataset characteristics

Author, Year	Country	Setting	Sample size	Report types	Train/Val/Test Split	Funding
Kwok, 2025^23^	Canada	Providence Health, BC	1795	Synoptic pathology reports	80%/10%/10%	CFI JELF, UBC
Balasubramanian, 2025^24^	USA/UK	BCN Generations Study, UK	111	Histopathology reports (scanned TIFF)	25% (Train)/75% (Test)	US NCI, Breast Cancer Now, ICR
Choi, 2023^25^	South Korea	Seoul National University Hospital	2931	Surgical pathology, ultrasound	NR (prompt engineering)	National Cancer Center (NCC), Ministry of Science and Information & Communication Technology
Hart, 2026^26^	USA	Mayo Clinic, Rochester	6651	DCIS, invasive carcinoma, phyllodes	NA (evaluation only)	Not specified
Sushil, 2024^27^	USA	UCSF Clinical Data Warehouse	769	Pathology and cytology reports	570/99/100	NIH/NCI
Cheligeer, 2024^28^	Canada	Four tertiary hospitals, Calgary	351	Surgical pathology (post-NAC)	80% (Train)/20% (Test) (5-fold CV)	CIHR
Liu, 2025^25^	USA	UCSF	978	Breast pathology reports	40%/30%/30%	Not specified
Bartels, 2025^26^	Germany	UMC Hamburg-Eppendorf	522^a^	Oncological pathology (German)	Bootstrapping (*n* = 1000)	Open Access Publication Fund of UKE - Universitätsklinikum Hamburg-Eppendorf
Hein, 2025^27^	USA	UT Southwestern	53	TCGA breast pathology	Iterative + external	Jan and Bob Pickens Distinguished Professorship in Medical Science and Brock Fund for Medical Science Chair in Pathology

*BCN* Breast Cancer Now, *BC* British Columbia, *NAC* neoadjuvant chemotherapy, *TCGA* The Cancer Genome Atlas, *NCI* National Cancer Institute, *ICR* Institute of Cancer Research, *CFI JELF* Canada Foundation for Innovation, *UBC* University of British Columbia, *CIHR* Canadian Institutes of Health Research, *CV* cross-validation, *NR* not reported, *NA* not applicable.

Sample size refers to the total number of pathology reports evaluated unless otherwise specified. For Choi et al.^25^, the sample size reflects the number of patients, each contributing one or more reports. Patient-level counts were not consistently reported across studies.

^a^Mixed oncological cohort; breast cancer cases represent ~7% of total sample.

### Model architectures and training approaches

The included studies evaluated over 30 distinct LLM architectures spanning multiple model families. GPT-family models were evaluated in six studies, including GPT-3.5-turbo^21,23^, GPT-4^23,25^, and GPT-4o variants^20,27^. Open-source alternatives were extensively represented, with Llama variants evaluated in five studies ranging from 8 billion to 405 billion parameters^20,22,25–27^. BERT-based architectures including ClinicalBERT, PubMedBERT, BioClinicalBERT, and BioMedRoBERTa were evaluated in three studies^19,23,24^. Emerging architectures including Gemini^22^, Mistral^19,26^, DeepSeek^22,25^, Qwen^27^, and Gemma^22^ were assessed in recent publications.

Training approaches varied substantially across studies. Zero-shot prompting without task-specific training was employed in five studies^20–23,27^, relying on instruction-tuned models’ inherent capabilities. Fine-tuning approaches were used in four studies^19,24–26^, typically employing parameter-efficient methods such as Low-Rank Adaptation (LoRA) to adapt pretrained models with limited computational resources^24,25^. Few-shot prompting with exemplar demonstrations was evaluated in two studies^26,27^. Retrieval-augmented generation combining LLMs with semantic search over reference documents was specifically examined by Bartels et al.^26^, demonstrating substantial performance improvements for smaller models. Table 2 summarizes model specifications and methodological approaches.

**Table 2:** Model specifications and methods

Author, Year	LLM architecture(s)	Parameter count	Training approach	Task formulation	Target data elements	Reference standard	Validation type
Kwok, 2025^23^	ClinicalBERT, PubMedBERT, BioMedRoBERTa, Mistral-Nemo	12.2B (Mistral)	Manual Prompt-tuning; Zero-shot	Extractive QA (SQuAD format); 3 FOI types: free text (13), categorical single (16), categorical multiple (3)	32 FOIs: tumor size, site, focality, histologic type, margins (invasive/DCIS), LN (examined, macro/micrometastases), Nottingham grade, nuclear grade, mitotic rate, LVI, necrosis, pathologic stage	Human annotation; IAA > 99%	Internal (train-test)
Balasubramanian, 2025^24^	GPT-4o, GPT-4o mini, Llama 3.1 (405B, 70B, 8B)	8B to 405B	Zero-shot prompting	Generative JSON extraction (structured output with strict formatting constraints)	51 features: specimen characteristics, lesion type, morphological/architectural features, tumor size, margin assessments, lymphovascular status, molecular scoring, grading, TNM staging	Conflict resolution (GPT-4o + human annotator); physician-adjudicated disagreements; human-vs-gold IAA 95.4%	Internal (train-test)
Choi, 2023^25^	GPT-3.5-turbo	Not specified	Zero-shot + prompt engineering	Generative extraction with two-step processing (Extract then Format functions)	From pathology: pT, pN, histologic grade, surgery type, NAC status, margin, ER, HER2, Ki-67, triple-negative, LVI, extracapsular extension, LN ratio, LN sampling, metastatic LN count. From ultrasound: nodule size, laterality, position, clinical T/N stage	Physician extraction; IAA NR	Internal (random sample)
Hart, 2026^26^	Gemini-2.5-pro, Gemini-2.0-flash, Llama3.3-70B, Phi4, DeepSeek-R1, Gemma3	14B to 70B	Off-the-shelf	Generative JSON with ontology mapping; agent-based multi-field extraction	229 discrete fields in 8 sections: specimen, tumor, margins, regional LN, distant metastasis, pTNM, additional findings, comments (86 subsections)	Expert annotation; IAA NR	Dual (synthetic + real)
Sushil, 2024^27^	GPT-4, GPT-3.5, Starling-7B, ClinicalCamel-70B	7B to 70B	Zero-shot (LLMs)	Document-level classification (9 single-label + 3 multi-label tasks); worst-prognosis focus for multiple features	12 tasks: specimen type, ER, PR, HER2, tumor grade, LN involvement, LVI, margin status, DCIS margin, tumor histology, sites examined, sites of disease	Oncology fellows; Krippendorff α = 0.85	Internal (held-out)
Cheligeer, 2024^28^	BERT variants, BART, FLAN-T5, GPT-2 (fine-tuned)	Not specified	Fine-tuning with LoRA	Binary document classification (pCR present vs. absent)	Single element: pathologic complete response (pCR) — absence of residual invasive malignant cells	Radiology fellow; κ = 1.0	Internal (5-fold CV)
Liu, 2025^25^	Llama-3.1 8B (fine-tuned); GPT-4, PMC-Llama; Mistral-7B-Instruct-v0.3; Gemma-2-9b-it	7B to 13B	LoRA fine-tuning	Hybrid: QA with structured JSON output; question-specific extraction	Breast: anatomic site, diagnosis (cancer/high-risk/benign), laterality-specific subtyping (DCIS, IDC, ILC, adenocarcinoma)	Trained Medical students; Cohen’s κ = 0.87	Internal (held-out)
Bartels, 2025^26^	Llama 3.3 70B, Mistral Small 24B, SauerkrautLM variants	8B to 70B	Zero-shot, Few-shot, RAG	NER with structured JSON output (Pydantic classes); RAG with semantic search over 15,000 reports	13 features: ICD-10, ICD-O topography/morphology, tumor grade, T/N/M categories, vascular invasion, perineural invasion, LVI, residual tumor, UICC stage	Oncology professionals; Cohen’s κ = 0.91	Internal (bootstrap)
Hein, 2025^27^	GPT-4o; Llama 3.3 70B, Qwen2.5 72B	70B to 72B	Zero-shot + iterative refinement	End-to-end generative extraction: entity identification, terminology normalization, relationship mapping, structured output	Breast (external): HER2 (IHC + FISH), ER, PR. Kidney (primary): histology subtypes, diagnosis, IHC/FISH (30+ assays), procedure, site	Pathologist adjudication	Internal + External (TCGA)

Task formulation and target data elements varied substantially across studies, reflecting fundamentally different extraction paradigms. BERT-based models were fine-tuned for structured prediction (extractive QA or classification), while autoregressive LLMs were applied in generative settings (JSON generation, ontology mapping). Performance comparisons across studies are sensitive to these differences, and readers should interpret results within the context of each study’s specific task formulation.

*IAA* inter-annotator agreement, *CV* cross-validation, *LoRA* Low-Rank Adaptation, *RAG* retrieval-augmented generation, *TCGA* The Cancer Genome Atlas, *NR* not reported, *FOI* field of interest, *QA* question answering, *JSON* JavaScript Object Notation, *pCR* pathologic complete response, *NAC* neoadjuvant chemotherapy, *DCIS* ductal carcinoma in situ, *IDC* invasive ductal carcinoma, *ILC* invasive lobular carcinoma, *IHC* immunohistochemistry, *FISH* fluorescence in situ hybridization, *SMOTE* Synthetic Minority Over-sampling Technique, *UICC* Union for International Cancer Control.

### Clinical data elements extracted

Studies targeted diverse clinical data elements spanning the breadth of pathology report content. Tumor characteristics including size, histologic type, and grade were extracted in seven studies^19–23,25,26^. Biomarker status including estrogen receptor, progesterone receptor, HER2 status, and Ki-67 index was targeted in six studies^20,21,23,25–27^. Margin assessment was evaluated in five studies^19–23^. Lymph node involvement including number examined, number positive, and presence of metastases was extracted in six studies^19–23,26^. TNM staging components were targeted in five studies^20–22,26,27^. Additional elements included lymphovascular invasion^21,23,26^, perineural invasion^26^, molecular markers^20,27^, and composite assessments such as pathologic complete response^24^.

Task formulation varied across studies. Document-level classification assigned categorical labels to entire reports^23,24^. Named entity recognition identified specific mentions within text^26^. Extractive question-answering located answer spans in response to structured queries^19^. Generative approaches produced structured outputs including JSON-formatted extractions^20,22,27^. Several studies employed hybrid approaches combining multiple formulations for different data element types^21,25^.

### Performance outcomes

Performance metrics varied across studies but demonstrated generally strong results for best-performing models. Overall accuracy for top models ranged from 87.7%^21^ to 97.4%^19^ across studies. These figures should not be interpreted as comparable across studies, as they reflect different task formulations (document classification, NER, extractive QA, generative extraction), different target data elements of varying complexity, different metric definitions (exact match vs. field-level vs. macro-averaged accuracy), and different report formats (synoptic vs. narrative vs. scanned). A structured summary of what each accuracy figure specifically measures is provided in Supplementary Table S6. F1-scores where reported ranged from 0.86^23^ to 0.97^26^. The highest reported accuracy of 97.4% was achieved by PubMedBERT fine-tuned on question-answering data for synoptic report extraction^19^. GPT-4 variants achieved accuracy of 96.1% in zero-shot settings, comparable to human annotator performance of 95.4%^20^.

Model architecture significantly influenced performance. Among studies directly comparing architectures, larger models generally outperformed smaller variants, though with diminishing returns at the highest parameter counts^20,22^. GPT-4o achieved 96.1% accuracy compared to 75.0% for Llama 3.1 8B in the same evaluation^20^. However, fine-tuned smaller models could approach or exceed zero-shot performance of larger models; Liu et al.^25^ demonstrated that fine-tuned Llama-3.1 8B achieved 91.5% accuracy compared to 84.0% for zero-shot GPT-4 on the same task.

Domain adaptation substantially improved performance. PubMedBERT pretrained on biomedical literature outperformed ClinicalBERT and general BERT variants^19^. Retrieval-augmented generation provided the most dramatic improvements for smaller models, with Llama 3.1 8B improving from 62% to 82% accuracy with RAG enhancement in the Bartels et al. study^26^; however, these figures reflect mixed-cohort performance and cannot be attributed specifically to breast cancer extraction given the small breast cancer subsample (*n* ≈ 37). Performance varied substantially across clinical data elements, with structured fields achieving highest performance (>95%) while complex interpretive elements showed greater variability; tumor location extraction achieved only 63.8% accuracy due to challenges with spatial reasoning^21^. Table 3 presents detailed performance outcomes.

**Table 3:** Performance outcomes

Author, year	Best model(s)	Accuracy	F1-score	Sensitivity	Precision	Comparator performance	Metric definition	Cost per report
Kwok, 2025^23^	PubMedBERT + SQuAD	97.4% (EM)	97.7%	NR	NR	ClinicalBERT 95.7%; BioMedRoBERTa 89.4%; Mistral 81.4%	Exact match (EM): extracted span must exactly match gold-standard answer; overall across 32 FOIs (answerable + unanswerable)	NR
Balasubramanian, 2025^24^	GPT-4o	96.1%	NR	NR	NR	Human annotator 95.4% (vs. gold standard); Llama 405B 94.7%; Llama 70B 91.6%; GPT-4o mini 87.9%; Llama 8B 75.0%	Field-level agreement with gold standard: percentage of individual extractions matching across 4232 total extractions (51 features × reports)	$0.44 (GPT-4o); $0.01 (GPT-4o mini)
Choi, 2023^25^	GPT-3.5-turbo	87.7%	NR	NR	NR	Range 47.6–98.2% by element, Average accuracy 87.7%	Average of element-specific accuracies across all targeted factors; range 47.6–98.2% by element (340 patient validation sample)	$0.03 per patient ($95.4 total for 2931)
Hart, 2026^26^	Gemini-2.5-pro	Syn 99.0%; Real 87.7%	98.3%	97.8%	98.9%	Gemini-flash 83.7%; Llama 61.8%	Synthetic: classification accuracy on 864 curated test cases (445 positive, 419 negative). Real: TP/(TP + FN) on 6,651 field annotations (no negative annotations)	NR
Sushil, 2024^27^	GPT-4 (zero-shot)	NR	0.86 (macro)	NR	NR	LSTM-Att 0.74; GPT-3.5 0.55; ClinicalCamel-70B 0.34	Macro-averaged F1 across 12 classification tasks (9 single-label, 3 multi-label), weighting each class equally	NR
Cheligeer, 2024^28^	GPT-2 Fine-tuned	95.8% (90.1–100%)	93.0% (83.7–100.0%)	95.3% (84.0–100.0%)	90.9% (76.5–100.0%)	GPT-2 Large 94.4%; FLAN-T5 small 80.3%	Binary classification accuracy for pCR determination (present/absent); 5-fold stratified CV with 10,000 bootstrap resamples for CI	NR
Liu, 2025^25^	Llama-3.1 8B (fine-tuned)	91.5% (88.1–94.6%)	NR	NR	NR	GPT-4 84.0%; Human 75.5%; PMC-Llama 4.8%	Dataset-level exact match accuracy: percentage of reports where all extracted fields match gold standard (breast-specific)	<$3 per model training; desktop GPU
Bartels, 2025^26^^a^	Llama 3.3 70B + RAG	97%	97%	98%	95–96%	Zero-shot F1 0.93; 8B + RAG 0.84	Macro-averaged accuracy across 13 features on 100 bootstrapped reports (*n* = 1000 iterations); stratified resampling	NR
Hein, 2025^27^	GPT-4o	89% (TCGA); 84.1% (EM)	NR	NR	NR	Qwen2.5 78.1%; Llama 70.1%	TCGA: agreement with curated tabular data for HER2/ER/PR (53 reports). Dev set: exact match across all extracted entities per report (152 reports)	NR

Confidence intervals shown where reported in original studies.

*EM* exact match, *NR* not reported, *Syn* synthetic validation, *pCR* pathologic complete response, *FOI* field of interest, *TP* true positive, *FN* false negative, *CV* cross-validation, *RAG* retrieval-augmented generation, *TCGA* The Cancer Genome Atlas, *LVI* lymphovascular invasion, *NAC* neoadjuvant chemotherapy. Note: Confidence intervals shown where reported in original studies.

^a^Performance figures reflect a mixed oncological cohort; breast cancer cases represent ~7% of the sample (*n* ≈ 37). These figures should not be interpreted as breast-cancer-specific extraction accuracy.

Performance figures are not directly comparable across studies due to fundamental differences in: (1) task formulation—extractive QA, document classification, binary classification, generative JSON extraction, and NER represent distinct computational tasks with different baseline difficulty levels; (2) target data elements—ranging from a single binary outcome (pCR) to 229 discrete fields, with simple structured fields (e.g., biomarker positive/negative) being inherently more tractable than complex interpretive elements (e.g., tumor location requiring spatial reasoning); (3) metric definitions—exact match, field-level accuracy, macro-averaged F1, and classification accuracy measure different aspects of performance; (4) report formats—synoptic reports with pre-structured fields yield higher accuracy than narrative or scanned reports; and (5) reference standard quality—IAA ranged from unreported to >99%, directly affecting the ceiling against which models are evaluated. Each row should be interpreted independently within its specific evaluation context. A detailed breakdown of what each accuracy figure measures is provided in Supplementary Table S6.

Interpretation of performance outcomes requires careful consideration of task formulation heterogeneity across studies. BERT-based models were typically fine-tuned for sequence labeling or token-level classification tasks optimized for structured prediction,¹⁵‘²⁰ whereas autoregressive LLMs such as GPT-4 were applied in generative settings framed as question answering or structured JSON generation.¹⁶‘¹⁸‘²³ These paradigms present fundamentally different challenges, and performance metrics are not directly comparable across task types. Furthermore, data element complexity varied substantially: structured fields such as biomarker status represent relatively straightforward span extraction, while composite variables such as TNM staging or pathologic complete response require contextual reasoning across multiple report sections. The observed performance variation across data elements likely reflects both model capability and inherent task difficulty, and future studies should report element-level performance stratified by task formulation to enable meaningful cross-study comparison.

### Methodological quality: PROBAST + AI assessment

The tables below present the PROBAST + AI plot showing quality assessment (model development) and risk of bias assessment (model evaluation) across all four domains. Overall, five studies (55.6%) achieved low concern/risk across all domains^19,23–26^, three studies (33.3%) had unclear ratings in at least one domain^20,22,27^, and one study (11.1%) was rated high concern^21^.

**Figure:**
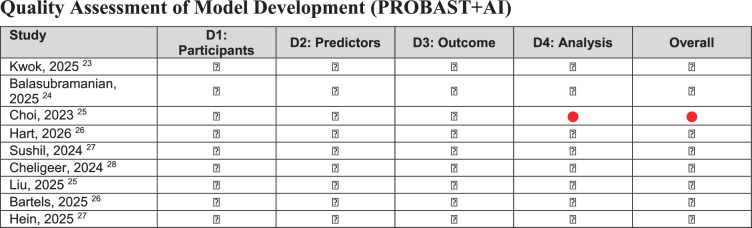

**Figure:**
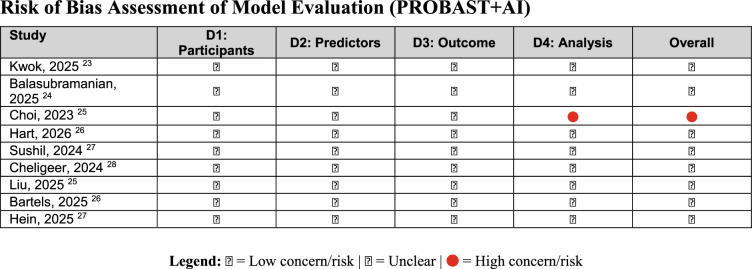

In Domain 1 (Participants and Data Sources) and Domain 2 (Predictors), all nine studies (100%) demonstrated low concern for quality and low risk of bias. Studies employed diverse but well-defined participant selection strategies including population-based cohorts, academic medical center data, and research cohorts. Clinical data elements were clearly defined with detailed data dictionaries or extraction schemas.

Domain 3 (Outcome) showed the greatest variability, with five studies (55.6%) rated low and four (44.4%) rated unclear. Studies with low concern demonstrated robust reference standard development: Kwok et al.^23^ achieved >99% inter-annotator agreement through iterative double annotation; Sushil et al.^27^ employed multiple annotators with Krippendorff’s alpha of 0.85; Cheligeer et al.^28^ reported perfect agreement (kappa = 1.0); Bartels et al.^26^ achieved Cohen’s kappa of 0.91 with two-step oncology-trained review. Four studies were rated unclear due to reference standard concerns: Balasubramanian et al.^24^ developed their gold standard through conflict resolution between GPT-4o and a single human annotator, introducing potential incorporation bias where the model being evaluated contributed to its own reference standard. Hart et al.^26^ and Hein et al.^27^ employed expert annotators but did not report formal inter-annotator agreement metrics. Choi et al.^25^ relied on single annotator with physician verification but no formal agreement measurement.

In Domain 4 (Analysis), seven studies (77.8%) demonstrated low concern/risk. Key considerations per PROBAST + AI signaling questions included avoiding evaluation based solely on apparent performance, reasonable sample sizes, appropriate missing data handling, proper class imbalance methods, avoiding data leakage between train/test sets, and comprehensive performance evaluation. Choi et al.^25^ was rated high concern due to absence of formal train/test separation and potential data leakage. Hart et al.^26^ was rated unclear due to the acknowledged ‘reality gap’ between synthetic and real-world performance (11–32 percentage point accuracy drops), though their dual validation strategy partially addressed internal validity.

### Reporting completeness: TRIPOD + AI assessment

TRIPOD + AI assessment revealed moderate reporting completeness with key gaps in areas emphasized by the updated guideline. Reporting strengths included participant eligibility criteria and predictor definitions (all studies fully reported) and data source descriptions (8/9 adequate). Seven studies provided sufficient analytical methods details.

**Fig. 2:**
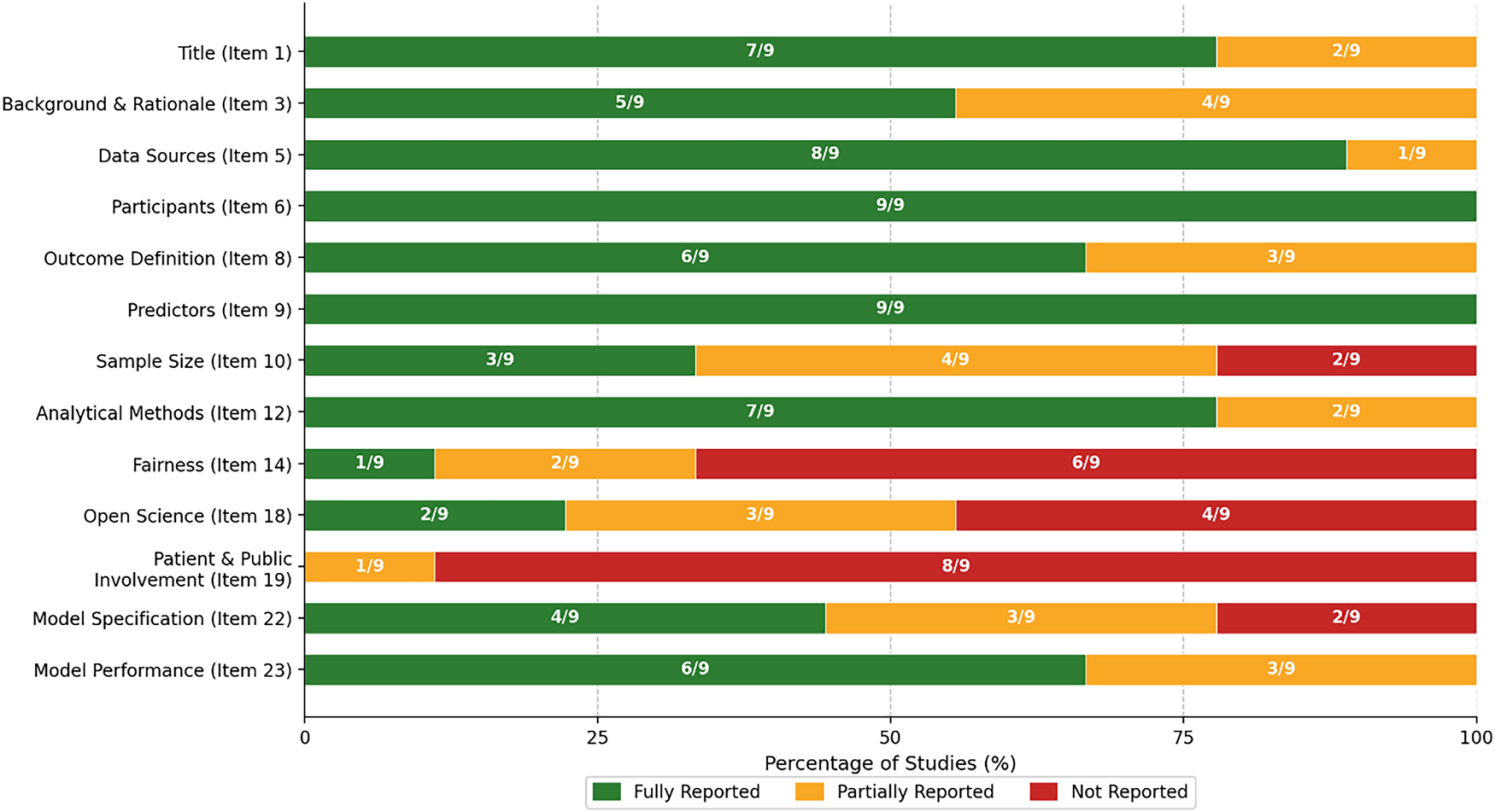
TRIPOD + AI reporting adherence across included studies. Horizontal stacked bar chart showing the percentage of studies achieving full (green), partial (yellow), or non-adherence (red) across key TRIPOD + AI reporting items. Participants (Item 6) and Predictors (Item 9) achieved universal full reporting across all nine studies, while Patient and Public Involvement (Item 19), Fairness (Item 14), and Open Science (Item 18) showed the greatest reporting deficits.

Critical reporting gaps were identified across three areas. First, fairness considerations (TRIPOD + AI item 14) were poorly addressed, with only one study fully reporting approaches to address model fairness and six studies not reporting fairness at all. Second, open science practices (item 18) were inconsistently reported: only four studies provided code availability^20,23,25,26^, and only two had registered protocols. Third, patient and public involvement (item 19) was rarely reported, with eight studies not mentioning PPI at all. Sample size justification (item 10) was inadequate in two studies. Notable exemplars included Bartels et al.^26^ and Sushil et al.^27^ for full code availability, Cheligeer et al.^28^ for following STARD reporting guidelines, and Liu et al.^25^ for comprehensive model specifications enabling third-party evaluation. Figure 2 presents the percentage of studies achieving full, partial, or non-adherence across key TRIPOD + AI items. Participants (Item 6) and Predictors (Item 9) achieved universal full reporting (9/9), while Patient and Public Involvement (Item 19), Fairness (Item 14), and Open Science (Item 18) showed the greatest reporting deficits. A detailed study-by-item matrix is provided in Supplementary Table S3.

## Discussion

This systematic review synthesizes evidence from nine studies^19–27^ evaluating LLM performance for extracting clinical data from breast cancer pathology reports, with rigorous methodological assessment using PROBAST + AI and TRIPOD + AI frameworks. The findings demonstrate that LLMs can achieve high accuracy for many extraction tasks, with best-performing models reaching 96–97% agreement with expert annotations^19,20,26^. This performance level approaches or exceeds human annotator consistency in several studies^20,25^, suggesting potential for meaningful automation of pathology report data extraction. However, important caveats temper enthusiasm: validation remains predominantly internal, the reality gap between controlled and clinical conditions is substantial^21^, and performance varies considerably across data elements and model configurations^20,25^.

The PROBAST + AI assessment revealed additional methodological concerns. While Participants/Data Sources and Predictors domains showed uniformly low concern (100%), the Outcome domain demonstrated substantial variability (44.4% unclear) primarily due to reference standard concerns. The use of single annotators without formal agreement metrics and, in one case, potential incorporation bias where the evaluated model contributed to gold-standard development^20^ represent methodological weaknesses that may inflate reported performance. The Analysis domain concern for one study^21^ regarding train/test separation highlights risks of data leakage in LLM evaluation studies.

The TRIPOD + AI assessment identified critical gaps in reporting practices newly emphasized for AI/ML prediction models. The near-absence of patient and public involvement reporting (8/9 studies) and limited fairness considerations (6/9 not reporting) represent significant gaps given increasing recognition that AI systems may perpetuate or amplify healthcare disparities^28^. Inconsistent adoption of open science practices limits reproducibility and third-party evaluation, undermining confidence in reported findings.

The evidence supports several key conclusions for clinical implementation. First, model selection matters significantly, with GPT-4 variants^23,26^ and fine-tuned domain-specific models^23,28^ consistently outperforming alternatives. Second, training approach influences outcomes, with fine-tuning providing substantial benefits over zero-shot application for many tasks^19,23,24^. Third, retrieval-augmented generation offers a practical path to improved performance without fine-tuning, particularly valuable for organizations unable to train custom models^26^. Fourth, performance varies by extraction target, with structured biomarker fields proving more tractable than complex interpretive elements requiring clinical reasoning^20,22^.

These findings align with broader trends in clinical NLP while highlighting domain-specific considerations. Prior systematic reviews of clinical information extraction have documented steady improvement from rule-based systems through machine learning to deep learning approaches^6,8^. Recent reviews have also examined LLM applications in breast cancer diagnosis more broadly, including Ahmed and Abbas who reviewed LLM techniques and outcomes across diagnostic tasks, further underscoring the growing evidence base in this domain. The performance levels observed for LLMs in breast cancer pathology extraction exceed those reported for earlier methods, consistent with the architectural advances enabling better contextual understanding^15^.

The observed reality gap between synthetic and real-world validation warrants particular attention. Hart et al.^22^ documented accuracy drops of 11–32 percentage points when moving from controlled test cases to clinical documents, a phenomenon consistent with findings in other clinical AI applications^28^. This gap likely reflects the difference between carefully curated evaluation sets and the messy reality of clinical documentation. Combined with the predominantly internal validation approaches identified (only one study with external validation^27^), published performance metrics should be interpreted cautiously when planning clinical deployment.

The review findings have direct implications for healthcare organizations considering LLM adoption for pathology report processing. For cancer registries and quality programs requiring high-volume data extraction, the evidence supports feasibility of LLM-assisted workflows for common data elements with established high performance^19,20,26^. Biomarker status, tumor grade, and margin assessments appear suitable for automated extraction with human verification of flagged cases. However, complex elements requiring clinical interpretation or spatial reasoning may require continued manual abstraction^21^.

Implementation planning should account for the substantial performance variation across models and configurations. Organizations with technical capacity for fine-tuning may achieve superior performance with open-source models adapted to local documentation patterns^19,25^. Those relying on commercial APIs should expect strong baseline performance from GPT-4 variants but should validate on local data before production deployment^20,23^. The cost-performance tradeoffs documented provide initial guidance, with per-report costs ranging from ~$0.01 to $0.50 based on API pricing at the time of each study’s publication^16,22^. These estimates reflect pricing available during 2023–2025 and may not reflect current API costs, which are subject to frequent revision by model providers.

The absence of external validation across institutions represents a critical gap for implementation. Organizations should approach published performance claims with appropriate skepticism and invest in local validation studies before clinical deployment. The documented variation in pathology reporting styles across institutions^21,25^ suggests that models performing well in one setting may require adaptation for others. Prospective monitoring of extraction accuracy should be built into any deployment strategy.

Several research priorities emerge from this synthesis. Most urgently, the field requires standardized evaluation frameworks enabling meaningful comparison across studies. Current heterogeneity in datasets, metrics, and validation approaches limits ability to draw definitive conclusions about optimal approaches^8^. Development of benchmark datasets representing diverse pathology reporting styles would enable rigorous head-to-head evaluation. Other research priorities also emerge from this synthesis informed by PROBAST + AI and TRIPOD + AI assessments. Most critically, future studies should establish robust reference standards with multiple annotators and formal agreement metrics, avoiding incorporation bias where evaluated models contribute to gold-standard development. External validation across institutions is essential, with only one study^27^ conducting such validation in the current evidence base. Cheligeer et al.^28^ drew data from four tertiary hospitals and a population-based registry, which could be considered multi-site validation; however, we classified this as internal validation because all sites were within a single regional health system and the model was trained and tested on pooled data rather than evaluated on held-out sites. Our classification criteria defined external validation as evaluation on data from institutions not represented in the training set. Multi-institutional collaborations should evaluate model generalization across different healthcare systems, pathology practices, and patient populations. The observed reality gap^21^ suggests particular attention to edge cases, unusual presentations, and documentation quality variation. Temporal validation examining performance stability as reporting practices evolve would address deployment sustainability concerns.

Adoption of TRIPOD + AI reporting guidelines would substantially improve evidence quality. Studies should explicitly address fairness considerations including performance evaluation across sociodemographic subgroups, adopt open science practices including protocol registration and code sharing, and consider patient and public involvement in study design and implementation where appropriate.

Research on implementation considerations beyond accuracy deserves greater attention. The limited reporting of cost, computational requirements, and workflow integration in current studies^19,20,24^ provides insufficient guidance for practical deployment. Studies examining human-AI collaboration models, appropriate levels of automation for different use cases, and change management strategies would support responsible implementation. Investigation of bias and fairness in LLM extraction across demographic groups represents an important but unexplored domain^22^.

This review employed rigorous systematic methodology including comprehensive database searching, dual independent screening, and standardized quality assessment using the contemporary PROBAST + AI and TRIPOD + AI frameworks^16–18^. The inclusion of computer science databases and preprint servers captured the rapidly evolving LLM literature. The multidisciplinary review team ensured appropriate evaluation of both technical and clinical aspects.

Several limitations merit acknowledgment. The rapid pace of LLM development means findings may not reflect the latest model capabilities. Our search was conducted through December 2025; given the emergence of newer model families since our search date, additional relevant studies may now exist. We do not believe this materially affects our conclusions regarding methodological quality and reporting gaps, but readers should note that performance benchmarks for the newest models may exceed those synthesized here. The predominance of English-language studies from high-income countries^19–25,27^ limits generalizability, with only one study examining non-English reports^26^. Both PROBAST + AI and TRIPOD + AI were designed primarily for prediction models and required adaptation for information extraction tasks. We acknowledge the recent publication of TRIPOD-LLM (Gallifant et al. 2025), which extends the TRIPOD framework specifically for studies using LLMs, broadening scope beyond prediction to encompass generative AI applications. Our protocol was registered and quality assessment commenced prior to the availability of TRIPOD-LLM; we therefore retained TRIPOD + AI for consistency with our registered methods. Future systematic reviews in this domain should consider adopting TRIPOD-LLM, which may better capture LLM-specific reporting elements such as prompt engineering documentation, temperature settings, and model versioning. We provide a brief comparison of TRIPOD + AI and TRIPOD-LLM coverage in Supplementary Table S4 to aid interpretation of our findings. The distinction between model development and evaluation is less clear-cut for zero-shot LLM approaches where no explicit training occurs. Heterogeneity in evaluation methods precluded formal meta-analysis.

The small number of included studies and heterogeneity in metrics precluded formal publication bias assessment. Given the rapid growth and high novelty value of LLM research, positive result bias is plausible, studies demonstrating poor LLM performance may be less likely to be published or may frame results differently. Our inclusion of preprint servers and gray literature partially mitigates but does not eliminate this concern, and readers should consider that the performance estimates synthesized here may represent an optimistic picture of the field.

The field stands at an inflection point where technical capability has largely been demonstrated but implementation infrastructure remains underdeveloped. Future progress requires movement from proof-of-concept studies to rigorous clinical validation, development of regulatory frameworks for AI-assisted data extraction, and establishment of quality assurance standards for deployed systems. Collaboration between AI researchers, clinical informaticists, pathologists, and implementation scientists will be essential for realizing the potential of LLMs to transform pathology report processing while maintaining accuracy and patient safety.

Emerging technical approaches warrant continued investigation. Multimodal models capable of processing both pathology report text and associated histopathology images may enable more comprehensive extraction^27^. Smaller, efficient models suitable for edge deployment could address privacy concerns about cloud-based processing^24,25^. Continued improvement in reasoning capabilities may address current limitations in complex interpretive extraction tasks^20,21^.

LLMs demonstrate promising performance for extracting clinical data from breast cancer pathology reports, with best-performing models achieving accuracy comparable to human experts^19,20,24,25^. GPT-4 variants and fine-tuned domain-specific models show superior performance,^19,23,24^ while retrieval-augmented generation offers practical improvements for resource-constrained settings^26^. However, the evidence base remains limited by predominantly internal validation,^19–27^ substantial reality gaps between controlled and clinical conditions^21^, and inadequate assessment of implementation considerations. Healthcare organizations should approach deployment with appropriate caution, investing in local validation and monitoring strategies. Future research should prioritize multi-institutional external validation, standardized evaluation frameworks, and investigation of deployment requirements to enable responsible clinical implementation of LLM-based pathology report extraction.

## Methods

### Study design and registration

This systematic review was conducted following the Preferred Reporting Items for Systematic Reviews and Meta-Analyses (PRISMA) 2020 guidelines^20^. The protocol was registered with PROSPERO prior to commencing the search (CRD420251012298). The multidisciplinary review team included expertise in medical informatics, clinical oncology, systematic review methodology, and NLP.

### Eligibility criteria

Studies were eligible if they evaluated LLM performance for extracting at least one clinical data element from breast cancer pathology reports. Eligible LLMs included any transformer-based architecture with documented parameter count exceeding 100 million, whether pretrained models used in zero-shot or few-shot settings, fine-tuned models adapted for pathology report extraction, or ensemble approaches combining multiple LLMs. For proprietary models with undisclosed parameter counts (e.g., GPT-3.5-turbo, GPT-4, GPT-4o), eligibility was inferred based on publicly available technical reports, independent estimates, and the models’ demonstrated capabilities on benchmark tasks, which are consistent with parameter counts substantially exceeding the 100 million threshold. We acknowledge this represents an inferential rather than documented criterion for these models, and future reviews may need to reconsider parameter-based eligibility thresholds as proprietary model architectures remain undisclosed. Studies examining extraction of any clinical data element including tumor characteristics, biomarker status, lymph node involvement, molecular markers, or composite elements were included. The reference standard required enabling performance assessment through manual annotation by clinical experts, comparison with structured database entries, or validation against other established extraction methods. Studies from any country or language were eligible without restrictions.

Exclusion criteria eliminated studies not providing specific evidence about LLM performance for breast cancer pathology reports. Studies examining only other cancer types without breast cancer data, those processing other report types without pathology reports, studies using only traditional NLP methods without LLM components, purely technical papers without clinical data extraction, and review articles without original performance data were excluded.

### Information sources and search strategy

The search strategy targeted databases covering biomedical, computer science, and artificial intelligence literature. Biomedical databases included MEDLINE (searched via Ovid) and PubMed (searched directly to capture ahead-of-print and non-MEDLINE indexed content), with Embase searched separately via Elsevier. Deduplication was performed in Covidence, which identified 251 duplicate records (see Fig. 1). Computer science databases comprised IEEE Xplore, ACL Anthology, and arXiv preprint server. Web of Science provided multidisciplinary coverage. The search strategy combined three concept blocks using Boolean operators: (1) breast cancer pathology (e.g., “breast neoplasms”[MeSH], “breast cancer”, “mammary carcinoma”, “pathology report*“, “histopath*“, “synoptic report*“); (2) large language models (e.g., “large language model*“, “LLM”, “GPT”, “BERT”, “transformer*“, “natural language processing”[MeSH], “deep learning”); and (3) information extraction (e.g., “information extraction”, “data extraction”, “text mining”, “named entity recognition”, “clinical coding”). Database-specific adaptations of the full search strings are provided in Supplementary Table S1. Supplementary searching included forward and backward citation tracking of included studies, gray literature screening of conference proceedings, and expert consultation.

### Study selection and data extraction

Study selection employed Covidence systematic review software. Two reviewers independently screened titles and abstracts followed by full-text review. Inter-reviewer agreement was assessed using Cohen’s kappa. Agreement was κ = 0.81 at title/abstract screening and κ = 0.77 at full-text review, the latter representing the lower end of substantial agreement. Disagreements at the full-text stage arose primarily around two criteria: the LLM parameter threshold for models with undisclosed parameter counts, and whether mixed-cohort studies provided sufficient breast-cancer-specific data to meet inclusion requirements. Disagreements were resolved through discussion, with a third reviewer (ZG) consulted when consensus could not be reached. For data extraction, agreement was assessed on a pilot set of three studies before proceeding to independent extraction of the remaining studies. Before commencing screening, reviewers completed calibration exercises, discussing disagreements to ensure consistent application of eligibility criteria. A standardized data extraction form captured: study characteristics (authors, year, country, setting, funding); dataset characteristics (sample size, report type, language, cancer subtype distribution, train/validation/test partition); model specifications (architecture name, parameter count, pretraining corpus, fine-tuning method); extraction task details (task formulation, target clinical data elements, prompt design); reference standard quality (number of annotators, expertise, inter-annotator agreement metric and value, adjudication process); validation methodology (internal, external, cross-validation, temporal); and performance outcomes (accuracy, F1-score, precision, recall, with confidence intervals where reported). The complete data extraction form is provided in Supplementary Table S2. Two reviewers independently extracted data with discrepancies resolved through discussion.

### Quality assessment

Methodological quality, risk of bias, and applicability were assessed using PROBAST + AI (Prediction model Risk Of Bias ASsessment Tool + Artificial Intelligence)^21^, published in 2025 by Moons et al. PROBAST + AI extends the original PROBAST framework to address AI/machine learning-based prediction models, including LLMs used for clinical information extraction. A key distinction in PROBAST + AI is the explicit separation between quality assessment (for model development) and risk of bias assessment (for model evaluation). PROBAST + AI comprises four domains: (1) Participants and Data Sources, (2) Predictors, (3) Outcome, and (4) Analysis.

Reporting completeness was assessed using TRIPOD + AI (Transparent Reporting of a multivariable prediction model for Individual Prognosis Or Diagnosis + AI)^22^, published in 2024 by Collins et al. TRIPOD + AI provides harmonized reporting guidance for prediction model studies regardless of methodology, with 27 main items covering title, abstract, introduction, methods, open science, patient and public involvement, results, and discussion. Two reviewers independently assessed each study using both frameworks; disagreements were resolved through discussion.

### Adaptation of assessment frameworks for information extraction tasks

Both PROBAST + AI and TRIPOD + AI were developed for clinical prediction models. Their application to information extraction tasks required systematic adaptation documented here and detailed in Supplementary Table S5. For PROBAST + AI, Domain 1 (Participants and Data Sources) signaling questions were applied without modification. Domain 2 (Predictors) questions regarding predictor definition were interpreted as referring to the input text representation and any preprocessing applied. Domain 3 (Outcome) questions were adapted such that ‘outcome definition’ was interpreted as the definition of extraction targets and reference standard construction, while ‘sample size for event rate’ was reinterpreted as the adequacy of positive examples for each extraction category. Domain 4 (Analysis) questions were applied with ‘calibration’ deemed not applicable for classification/extraction tasks. For TRIPOD + AI, items relating to outcome prediction horizons (items 6b, 12) were deemed not applicable; all other items were applied as specified. We considered alternative frameworks including QUADAS-2 for diagnostic accuracy; however, PROBAST + AI provided more comprehensive coverage of AI-specific methodological considerations including data leakage, class imbalance handling, and model development versus evaluation distinction.

### Assessment of reporting bias

We assessed the potential for reporting bias through several approaches. First, our search strategy included preprint servers (arXiv), conference proceedings (IEEE Xplore, ACL Anthology), and gray literature to capture studies irrespective of publication status. Second, we narratively assessed evidence of selective outcome reporting within included studies by comparing registered protocols (where available) with published results. Formal statistical assessment of publication bias (e.g., funnel plots, Egger’s test) was not feasible due to the small number of included studies (*n* = 9) and substantial heterogeneity in outcome metrics precluding pooling. The implications of potential publication bias are considered in the Discussion.

## Supplementary information

Supplementary Information

## Data Availability

All data supporting the findings of this systematic review are available within the article and its supplementary materials. No new datasets were generated during this study.

## Code availability

Not applicable. No custom code was developed for this systematic review.
