# Supplementary material for: Performance of large language models for extracting clinical data from breast cancer pathology reports: a systematic review
Source: NPJ Digit Med. 2026 May 22;9:627. doi: 10.1038/s41746-026-02789-x (PMC13473680; doi:10.1038/s41746-026-02789-x)
Supplement: Supplementary file 1 [file 41746_2026_2789_MOESM1_ESM.pdf]

Supplementary Table S1: Full Search Strategies for All Databases

Search conducted: from databases inception to December 2025. No date or language restrictions applied.

Database	Search String
**MEDLINE (via Ovid)**	1. exp Breast Neoplasms/ OR (breast cancer OR breast carcinoma OR breast neoplasm* OR mammary carcinoma).mp.<br>2. exp Pathology, Surgical/ OR (pathology report* OR patholog* report* OR histopath* OR synoptic report*).mp.<br>3. (large language model* OR LLM OR LLMs OR GPT OR GPT-4 OR GPT-3 OR BERT OR transformer* OR generative pre-trained).mp.<br>4. exp Natural Language Processing/ OR (natural language processing OR NLP OR text mining OR information extraction OR named entity recognition OR clinical NLP).mp.<br>5. 1 AND 2 AND (3 OR 4)<br>[Adapt MeSH terms and field tags per database]
**PubMed (direct)**	("breast neoplasms"[MeSH] OR "breast cancer" OR "breast carcinoma") AND ("pathology" OR "pathology report" OR "histopathology" OR "synoptic report") AND ("large language model" OR "LLM" OR "GPT" OR "BERT" OR "transformer" OR "natural language processing"[MeSH] OR "information extraction" OR "text mining")
**Embase (via Elsevier)**	('breast cancer'/exp OR 'breast neoplasm' OR 'breast carcinoma') AND ('pathology report' OR 'histopathology' OR 'synoptic report') AND ('large language model' OR 'LLM' OR 'GPT' OR 'BERT' OR 'transformer' OR 'natural language processing'/exp OR 'information extraction')
**IEEE Xplore**	("breast cancer" OR "breast neoplasm") AND ("pathology report" OR "histopathology") AND ("large language model" OR "LLM" OR "GPT" OR "BERT" OR "natural language processing" OR "information extraction")
**ACL Anthology**	breast cancer pathology large language model OR breast cancer NLP extraction OR pathology report transformer
**arXiv**	ti:("breast cancer") AND abs:("pathology report" OR "pathology") AND abs:("large language model" OR "LLM" OR "GPT" OR "BERT" OR "NLP" OR "information extraction")<br>Categories: cs.CL, cs.AI, cs.LG
**Web of Science**	TS=("breast cancer" OR "breast neoplasm") AND TS=("pathology report" OR "histopath*" OR "synoptic report") AND TS=("large language model*" OR "LLM" OR "GPT" OR "BERT" OR "natural language processing" OR "information extraction" OR "text mining")

Supplementary Table S2: Standardized Data Extraction Form

Category	Data Element	Description / Coding Instructions
**Study Characteristics**	First author, year	As reported
	Country	Country of study conduct
	Setting	Institution type (academic, community, registry, multi-site)
	Study design	Retrospective, prospective, or mixed
	Funding source	Government, industry, institutional, none, not reported
	Publication type	Peer-reviewed journal, preprint, conference paper
**Dataset Characteristics**	Sample size (total reports)	Total number of pathology reports in the study
	Sample size (test set)	Number of reports used for final performance evaluation
	Breast cancer subset (if mixed cohort)	Number and percentage of breast cancer-specific reports
	Report type	Surgical pathology, core biopsy, synoptic, scanned, cytology
	Report language	English, German, etc.
	Data source period	Date range of reports included
	Train/validation/test split	Proportions and absolute numbers for each partition
	Patient demographics	Age, sex, race/ethnicity distribution if reported
**Model Specifications**	Model architecture name(s)	Exact model identifiers (e.g., GPT-4o, Llama-3.1-8B)
	Parameter count	Number of parameters; "not disclosed" for proprietary models
	Pretraining corpus	General, biomedical, clinical; specific corpus name if reported
	Fine-tuning method	Full fine-tuning, LoRA, QLoRA, adapter-based, none
	Fine-tuning data size	Number of examples used for fine-tuning
	Prompting strategy	Zero-shot, few-shot (number of examples), chain-of-thought, instruction-based
	RAG implementation	Yes/No; if yes, retrieval method and knowledge base description
	Temperature and hyperparameters	Temperature, top-p, max tokens, other generation parameters
**Extraction Task Details**	Task formulation	Document classification, NER, extractive QA, generative JSON, hybrid
	Target data elements	List of all clinical variables extracted (e.g., ER, PR, HER2, tumor size, grade, margins, LN status, TNM, LVI)
	Number of target elements	Count of distinct data elements extracted
	Output format	Categorical labels, free text spans, structured JSON, other
**Reference Standard**	Annotator number and expertise	Number of annotators, clinical role (pathologist, oncologist, fellow, student)
	Annotation process	Independent dual annotation, single with verification, consensus, other
	Inter-annotator agreement metric	Cohen's kappa, Krippendorff's alpha, percent agreement, other
	Inter-annotator agreement value	Reported numeric value
	Adjudication process	Third reviewer, discussion, model-assisted, none

Category	Data Element	Description / Coding Instructions
	Potential incorporation bias	Whether the evaluated model contributed to reference standard development
**Validation Methodology**	Validation type	Internal (random split, cross-validation, bootstrap) or External (separate institution)
	External validation institution(s)	Name and geographic location of external validation site(s)
	Temporal validation	Whether data from different time periods was used for validation
**Performance Outcomes**	Primary metric	Accuracy, F1-score, AUC, or other primary metric reported
	Primary metric value (best model)	Numeric value with confidence interval if reported
	Metric definition	Exact match, field-level, macro-averaged, micro-averaged, weighted
	Element-level performance	Performance broken down by individual data element
	Comparator model performance	Performance of alternative models or baselines evaluated
	Human comparator performance	Human annotator/abstractor performance if reported
	Processing time / cost	Per-report processing time and/or API cost if reported
	Error analysis	Types and patterns of extraction errors described

Supplementary Table S3: TRIPOD+AI Item-Level Assessment Matrix

TRIPOD+AI Item	Kwok 2025	Balasubramanian 2025	Choi 2023	Hart 2026	Sushil 2024	Cheligeer 2024	Liu 2025	Bartels 2025	Hein 2025
**Title & Abstract**									
1. Title	Y	N	N	Y	Y	Y	Y	Y	Y
2. Abstract	Y	Y	P	P	Y	Y	Y	Y	Y
**Introduction**									
3a. Background and rationale	Y	Y	P	P	P	Y	Y	Y	P
3b. Objectives	Y	Y	Y	Y	Y	Y	Y	Y	Y
**Methods**									
4. Source of data	Y	Y	Y	Y	Y	Y	Y	Y	Y
5. Participants	Y	Y	P	Y	Y	Y	Y	Y	P
6a. Outcome definition	Y	Y	N	Y	N	Y	Y	Y	N
6b. Outcome timing	NA	NA	NA	NA	NA	NA	NA	NA	NA
7. Predictors	Y	Y	Y	Y	Y	Y	Y	Y	Y
8. Sample size	Y	Y	P	Y	Y	Y	Y	P	N
9. Missing data	P	N	N	P	Y	Y	P	P	N
10. Sample size justification	Y	Y	P	P	P	Y	P	P	N
11. Model development	Y	Y	P	Y	Y	Y	Y	Y	Y
12. Model specification	Y	Y	P	Y	Y	Y	Y	Y	Y
13. Model performance	Y	Y	Y	Y	Y	Y	Y	Y	Y
14. Fairness	N	P	N	N	N	N	N	N	Y
15. Model examination	P	P	N	P	Y	P	Y	Y	P
16. Sensitivity analyses	P	Y	N	Y	P	Y	P	Y	P
**Open Science & PPI**									
17. Protocol registration	N	N	N	N	N	Y	N	N	N
18. Code/data availability	N	Y	N	N	Y	N	Y	Y	N
19. Patient/public involvement	N	N	N	N	N	N	N	N	Y
**Results**									
20. Participants	Y	Y	Y	Y	Y	Y	Y	Y	Y
21. Model performance	Y	Y	Y	Y	Y	Y	Y	Y	Y
22. Model updating	NA	NA	NA	NA	NA	NA	NA	NA	NA
**Discussion**									
23. Interpretation	Y	Y	P	Y	Y	Y	Y	Y	Y
24. Implications	Y	Y	P	Y	Y	Y	Y	Y	Y
25. Limitations	Y	Y	P	Y	Y	Y	Y	Y	Y
**Other**									
26. Supplementary materials	P	Y	N	P	Y	Y	Y	Y	P
27. Funding	Y	Y	Y	N	Y	Y	N	Y	Y

TRIPOD+AI Item	Kwok 2025	Balasubramanian 2025	Choi 2023	Hart 2026	Sushil 2024	Cheligeer 2024	Liu 2025	Bartels 2025	Hein 2025
**Legend:**	Y = Fully Reported			P = Partially Reported			N = Not Reported		

Supplementary Table S4: Comparison of TRIPOD+AI and TRIPOD-LLM Frameworks

TRIPOD+AI: Collins et al. (2024), BMJ. TRIPOD-LLM: Gallifant et al. (2025), Nature Medicine.

Aspect	TRIPOD+AI (Used in This Review)	TRIPOD-LLM	Implications for This Review
**Scope**	Prediction models using regression or ML/AI methods	Any study using LLMs (prediction, generation, extraction, summarization, etc.)	TRIPOD-LLM has broader scope that better encompasses information extraction tasks; TRIPOD+AI required adaptation for non-prediction tasks
**Target models**	All AI/ML models including LLMs used for prediction	Specifically designed for LLMs regardless of task type	TRIPOD-LLM is more directly applicable to the generative LLMs evaluated in most included studies
**Prompt engineering reporting**	Not specifically addressed	Explicit items for prompt template documentation, prompt selection rationale, and prompt sensitivity analysis	A key gap: prompt engineering was central to most included studies but TRIPOD+AI does not require its structured reporting
**Temperature & generation parameters**	Covered under general model specification	Dedicated items for temperature, top-p, max tokens, and other generation hyperparameters	Only 3/9 studies reported temperature settings; TRIPOD-LLM would have flagged this gap more clearly
**Model versioning & access**	General reproducibility guidance	Specific items for model version, API version, access date, and checkpoint specification	Critical for proprietary models (GPT-4, GPT-4o) where behavior may change across API versions
**Hallucination & output verification**	Not specifically addressed	Items for hallucination assessment, output verification strategies, and factual consistency checking	Directly relevant: none of the included studies systematically assessed hallucination in extraction outputs
**Input/output format**	General predictor and outcome specification	Specific items for input format (structured, free text, multimodal) and output format specification	Would have enabled more systematic comparison of task formulations across studies
**Few-shot & in-context learning**	Not specifically addressed	Items for example selection, number of examples, and in-context learning strategy	Relevant to 2/9 studies using few-shot approaches; TRIPOD-LLM provides clearer reporting expectations
**Fine-tuning documentation**	Covered under model development	Expanded items for fine-tuning dataset description, training hyperparameters, convergence criteria	Would improve reproducibility for the 4/9 studies employing fine-tuning approaches
**Fairness & bias**	Single item on fairness considerations	Expanded coverage including demographic bias testing, training data representation, and performance disparities	Aligns with our finding that 6/9 studies did not report fairness; TRIPOD-LLM provides more granular guidance
**Cost reporting**	Not specifically required	Items for computational cost, API cost per inference, and resource requirements	Only 2/9 studies reported cost data; TRIPOD-LLM would mandate this reporting
**Publication date**	2024 (BMJ)	2025 (Nature Medicine)	TRIPOD-LLM was published after our protocol registration and quality assessment was underway

Supplementary Table S5: Adaptation of PROBAST+AI Signalling Questions for Information Extraction Tasks

Domain / Signalling Question	Original Focus (Prediction Models)	Adaptation for Information Extraction	Rationale
**DOMAIN 1: PARTICIPANTS AND DATA SOURCES**			
**1.1 Were appropriate data sources used?**	Clinical cohort representative of target population for prediction	Pathology report corpus representative of clinical documentation diversity (report types, formats, institutions)	Applied without major modification; "population" interpreted as the range of pathology reports the model would encounter in deployment
**1.2 Were appropriate inclusion/exclusion criteria applied?**	Clinical eligibility criteria for prediction cohort	Criteria for report selection (breast cancer-specific, language, format completeness)	Applied without modification
**1.3 Were participants selected appropriately?**	Avoidance of selection bias in clinical cohort	Avoidance of selection bias in report sampling (e.g., not limited to "clean" or easy reports)	Applied without modification; assessed whether studies selected convenience samples of well-formatted reports
**DOMAIN 2: PREDICTORS**			
**2.1 Were predictors defined and assessed appropriately?**	Clinical predictor variables measured reliably	Input text representation and preprocessing defined clearly (tokenization, text cleaning, OCR quality for scanned reports)	"Predictors" interpreted as the input features (text) provided to the model
**2.2 Were predictors available at intended time of use?**	Predictor availability at prediction time point	Report text available at time of extraction (not dependent on future documentation)	Applied without modification; all studies used retrospective report text
**2.3 Were all relevant predictors included?**	Inclusion of established clinical predictors	Inclusion of complete report text (not truncated or selectively excerpted)	Assessed whether input text was truncated due to context window limitations
**DOMAIN 3: OUTCOME**			
**3.1 Was the outcome defined appropriately?**	Clinical outcome with clear temporal definition	Extraction target (clinical data element) defined with clear coding scheme and permissible values	"Outcome" reinterpreted as the target extraction variable; "outcome definition" = data dictionary / extraction schema
**3.2 Was the outcome determined appropriately?**	Outcome ascertained without knowledge of predictors	Reference standard developed independently of model output; assessors blinded to model predictions	Applied without modification; specifically assessed incorporation bias where model outputs informed reference standard
**3.3 Was the outcome determined using the same method for all participants?**	Consistent outcome measurement across cohort	Same reference standard annotation process applied across all reports	Applied without modification
**3.4 Was the sample size adequate for event rate?**	Sufficient outcome events for stable prediction	Sufficient positive examples for each extraction category (e.g., adequate number of HER2-positive cases)	"Event rate" reinterpreted as class prevalence for each extraction target; assessed whether rare categories had sufficient representation
**3.5 Was the time interval appropriate?**	Appropriate follow-up for outcome ascertainment	Deemed not applicable for extraction tasks	No temporal component in information extraction; marked NA for all studies
**DOMAIN 4: ANALYSIS**			
**4.1 Were appropriate performance measures used?**	Discrimination, calibration, clinical utility	Appropriate extraction metrics: accuracy, F1-score, precision, recall; exact match vs. partial match	"Calibration" deemed not applicable for classification/extraction tasks; assessed whether metrics matched task requirements
**4.2 Was model overfitting addressed?**	Cross-validation, regularization, independent test set	Proper train/test separation, cross-validation, or held-out test set; avoidance of data leakage	Applied without modification; particularly relevant for fine-tuned models
**4.3 Was the sample size reasonable?**	Events per variable rule of thumb	Adequate test set size for stable performance estimation	Prediction-specific rules (e.g., 10 EPV) not applied; instead assessed whether

Domain / Signalling Question	Original Focus (Prediction Models)	Adaptation for Information Extraction	Rationale
			test set was large enough for meaningful confidence intervals
**4.4 Were missing data handled appropriately?**	Imputation or complete case analysis justified	Handling of reports with missing fields, incomplete reports, or ambiguous documentation	Applied with minor adaptation; "missing data" includes reports with missing sections or unclear field values
**4.5 Was model complexity appropriate?**	Parsimonious model for available sample size	Model size appropriate for training data available (particularly for fine-tuning)	Applied without modification; assessed whether fine-tuned models used sufficient training data relative to parameter count
**4.6 Was evaluation performed on appropriate data?**	Independent validation data	Test data representative of intended deployment setting	Applied without modification; assessed internal vs. external validation

Supplementary Table S6: Structured Summary of Performance Metrics Across Studies

Note: Performance figures are NOT directly comparable across studies. This table clarifies what each reported metric actually measures.

Study	Reported Best Accuracy/F1	Metric Definition	Task Formulation	Target Elements Evaluated	Report Format	Key Comparability Caveats
**Kwok, 2025**	97.4% (EM accuracy)	Exact match: extracted answer span must exactly match gold-standard answer	Extractive QA (SQuAD-format)	Tumor size, histologic type, grade, margins, LN status	Synoptic (structured) pathology reports	Synoptic format provides pre-structured fields; EM metric is strict; performance may differ on narrative reports
**Balasubramanian, 2025**	96.1% (field-level accuracy)	Percentage of individual data fields correctly extracted across all reports	Generative JSON extraction	ER, PR, HER2, tumor size, grade, margins, LN, TNM, molecular markers	Scanned TIFF histopathology reports	Includes OCR step; field-level accuracy averages across elements of varying difficulty; small sample (n=111)
**Choi, 2023**	87.7% (average accuracy)	Average of element-specific accuracies across all targeted data elements	Generative (prompt engineering)	Tumor size, grade, type, margins, LN, TNM, LVI, location	Surgical pathology + ultrasound reports	Averaging masks wide element-level variation (47.6%–98.2%); includes complex elements like tumor location (63.8%)
**Hart, 2026**	99.0% synthetic / 87.7% real	Field-level accuracy on synthetic (curated) vs. real clinical reports	Generative JSON (agent-based)	Tumor size, grade, type, margins, LN, TNM	Synoptic reports (DCIS, invasive, phyllodes)	Major "reality gap" (11–32 pp drop); synthetic accuracy inflates expectations; preprint (not peer reviewed)
**Sushil, 2024**	0.86 (macro F1)	Macro-averaged F1 across all classification categories, weighting each class equally	Document-level classification	ER, PR, HER2, histologic type, margins, LN, LVI	Pathology and cytology reports	Macro F1 penalizes poor performance on rare classes; includes both pathology and cytology; zero-shot only
**Cheligeer, 2024**	95.8% (accuracy)	Classification accuracy for binary pCR determination (yes/no)	Document classification (binary)	Pathologic complete response (pCR) only	Surgical pathology (post-NAC)	Single binary outcome (pCR); simpler task than multi-element extraction; 5-fold CV may have optimistic bias
**Liu, 2025**	91.5% (field-level accuracy)	Percentage of individual data fields correctly extracted; 95% CI: 88.1–94.6%	Hybrid (classification + generative)	ER, PR, HER2, tumor size, grade, histologic type	Breast pathology reports	Fine-tuned 8B model outperformed zero-shot GPT-4 (84.0%); demonstrates fine-tuning benefit but limited to single institution
**Bartels, 2025**	97% accuracy / 0.97 F1	Field-level accuracy and F1 for extracted attributes across mixed oncological cohort	NER + Generative (RAG-enhanced)	Tumor size, grade, biomarkers, LN, TNM, LVI, perineural invasion	German-language oncological pathology reports	Mixed cohort (only ~7% breast cancer ≈ 37 reports); German language; RAG-enhanced; breast-specific performance not disaggregated

Study	Reported Best Accuracy/F1	Metric Definition	Task Formulation	Target Elements Evaluated	Report Format	Key Comparability Caveats
**Hein, 2025**	89% (TCGA) / 84.1% (EM)	TCGA accuracy on external dataset; exact match on internal validation	Generative (iterative refinement)	TNM staging, biomarkers, molecular markers	TCGA breast pathology reports	Small external sample (n=53); iterative refinement approach; only study with true external validation
